# Research on the Influence of Media Internalized Pressure on College Students’ Sports Participation—Chained Intermediary Analysis of Social Physique Anxiety and Weight Control Self-Efficacy

**DOI:** 10.3389/fpsyg.2021.654690

**Published:** 2021-05-12

**Authors:** Yiyi Ouyang, Jiong Luo, Jinsheng Teng, Tingran Zhang, Kun Wang, Jing Li

**Affiliations:** ^1^Institute of Physical Education, Chongqing University of Posts and Telecommunications, Chongqing, China; ^2^Institute of Physical Education, Southwest University, Chongqing, China; ^3^Institute of Physical Education, Sichuan University, Chengdu, China

**Keywords:** media internalized pressure, social physique anxiety, weight control efficacy, sports participation, intermediary effect

## Abstract

**Purpose:** Discuss the relationship among college students’ media internalized pressure, social physique anxiety, weight control self-efficacy, and sports participation in providing a reference for promoting college students to develop healthy and confident living habits.

**Methods:** Take Southwest University in China as the object, select the subjects by stratified random sampling, and process the data with SPSS19.0 and AMOS21.0 statistical software.

**Results:** (1) Media internalized pressure is positively correlated with social physique anxiety, weight control self-efficacy, and sports participation; social physique anxiety is significantly positively correlated with weight control self-efficacy and sports participation, and weight control self-efficacy is significantly positively correlated with sports participation; (2) media internalized pressure has a direct effect on sports participation (ES = 0.456), and social physique anxiety (ES = 0.136) and weight control self-efficacy (ES = 0.102) play significant mediating roles in the relationship between media internalized pressure and sports participation, respectively; the chained mediating force of social physique anxiety and weight control self-efficacy also reaches a significant level (ES = 0.027).

**Conclusion:** Media internalized pressure can influence college students’ sports participation through the direct path as well as indirect paths such as social physique anxiety, the intermediary effect of weight control self-efficacy, and chained intermediary effect of social physique anxiety–weight control self-efficacy, and social physique anxiety is another key factor affecting college students’ sports participation except media internalized pressure.

## Introduction

Mass media refers to newspapers, magazines, television, radio, movies, books, audio-visual products, and the Internet and other media to spread social and cultural information (Wang et al., 2016). In recent years, with the rapid development of mass media, its functions and the service scope have also been greatly enhanced, especially the emerging social media platforms that are widely sought after by contemporary college students, playing an important role in their lives, as well as providing faster, vivid, and concise access to information (Hu et al., 2021). Some scholars have studied college students’ sports participation through mass media and found that college students can learn more about sports through emerging social media, which also has a promotional effect on sports and which in turn can enhance college students’ sports participation. Therefore, mass media has a promotional effect on college students’ physical health (Hang and Deng, 2017; Cao and Chen, 2020). However, some scholars have also explored the psychology of college students through the mass media and found that the mass media tends to portray the “ideal body shape” of male and female students according to their own position, and college students living in this sexually objectified environment are prone to self-objectification. They unconsciously accept this “inculcation” and transform it into their own thoughts through the process of internalization, examining their own bodies from the perspective of this objectification and appearing to self-objectify; thus, media internalized pressure is generated (Liang et al., 2020; Yang et al., 2020).

Media internalized pressure is divided into two parts: one is media internalization, and the other is media pressure. Media internalization refers to the psychological process by which individuals cognitively accept a certain ideal body shape rendered by the media and assimilate it into their worldview, life view, and values, and thus, this concept plays a leading role in their lives (Stice et al., 2000; Thompson and Stice, 2001; Dittmar and Howard, 2004). Media pressure refers to the pressure perceived from the media (Cusumano and Thompson, 1997; Thompson et al., 2004). Due to the role of mass media as a source of information about ideal appearance, college students are excessively exposed to media images of slim female models or muscular male models, which will lead to internalization of ideal beauty standards propagated by the media and perception of this ideal body state as real, universal, and approved (Hawkins et al., 2004; Qin, 2009; Galioto and Crowther, 2013). At the same time, mass media is often considered to be an important factor in causing individual body imagery disorders because the body shapes of models, celebrities, and actors conveyed in the media are difficult to achieve and attain, leading to reduced self-body satisfaction when the gap between the real body self and the ideal body self-produced under the influence of the media is large (Fallon, 1990; Groesz et al., 2002; Smeets et al., 2010; Ruhl et al., 2011). In addition, nowadays, college students are vulnerable to the influence of media internalized pressure, leading to physical dissatisfaction, and which has become a common phenomenon. So, will college students’ dissatisfaction with their bodies lead to a series of related psychological problems? Some studies have pointed out that social media creates a new esthetic standard, which will increase the media internalized pressure of college students. The longer the reading or watching time is, the greater the psychological pressure and negative body image will be, which will lead to social physique anxiety (Harrison, 2010; Sharp et al., 2014; Yang et al., 2020).

Social physique anxiety refers to a specific subtype of social anxiety that develops in response to the physique. In the expected or real social situation, individuals want to leave a good physique impression on others, but they doubt whether they have this ability (Bao et al., 2010; Xu et al., 2017). According to relevant study reports, excessive media internalized pressure will lead to the rise of negative body image and social physique anxiety among college students, which will cause them to change their physical behavior (Sharp et al., 2014; Tylka et al., 2015; Slater et al., 2017), and usually, college students choose to engage in sports to enhance body imagery (Gillen, 2015; Andrew et al., 2016; Ouyang et al., 2020); therefore, media internalized pressure is a contributor to college students’ sports participation. Meanwhile, some scholars believe that social physique anxiety can boost the degree of sports participation and that social physique anxiety is one of the motivations to drive sports participation (Eklund and Crawford, 1994; Hausenblas and Martin, 2000; He, 2018). This seems to have proved that media internalized pressure can have an effect on sports participation through social physique anxiety and whether it is also suggesting that social physique anxiety may mediate the relationship between media internalized pressure and sports participation.

Several studies on media internalized pressure have also suggested that media internalized pressure has a positive predictive effect on weight control self-efficacy. The higher the media internalized pressure, the better their weight control self-efficacy and *vice versa* (Chen et al., 2009; Chih and Chia, 2018). Weight control self-efficacy refers to the beliefs maintained about one’s ability to perform a specific task or assignment and is also the level of confidence that students are confident in weight control, which includes dietary self-efficacy and exercise self-efficacy (Sallis et al., 1988; Yung, 2016; Chih and Chia, 2018; Chin and Chih, 2019). Some studies have also found that weight control self-efficacy has a good positive predictive effect on sports participation (Kuan and Huang, 2012; Yea and Yea, 2014; Meng et al., 2017; Annesi, 2019; Shang and Ku, 2019). One study also found that media internalized pressure can have a negative effect on one’s body image satisfaction, and once one’s body image satisfaction decreases, weight control behaviors increase (Hsin and Hung, 2008; Sun et al., 2013; Cao et al., 2014), and it also contributes to weight control self-efficacy (Chen et al., 2009; Chih and Chia, 2018), which in turn can effectively increase college students’ sports participation. It seems that weight control self-efficacy may play a mediating role between media pressure and sports participation.

In addition, other studies have shown that social physique anxiety can also have a positive impact on weight control self-efficacy, and the improvement of social physique anxiety can promote the level of weight control self-efficacy (Shu et al., 2011; Annesi and Mareno, 2015; Yuan et al., 2016). In summary, media internalized pressure can have an impact on sports participation through social physique anxiety or weight control self-efficacy, but according to the current study, the relationship between media internalized pressure and sports participation was hardly explored through the social physique anxiety or the mediating role of weight control self-efficacy. Because there is also a positive correlation between social physique anxiety and weight control self-efficacy, will the media internalized pressure have an impact on college students’ sports participation through social physique anxiety and weight control self-efficacy? At the same time, could it be that social physique anxiety is elevated in college students as a result of media internalized pressure to develop stable weight control self-efficacy, and is it a key factor to promote sports participation? It is because very few scholars have explored the relationship between “media internalized pressure + social physique anxiety + weight control self-efficacy + sports participation,” especially the chain of “social physique anxiety + weight control self-efficacy.” There is a lack of in-depth research on the existence of the mediating mechanism of “social physique anxiety + weight control self-efficacy.”

Based on this, this study constructs a chain mediation model of media internalized pressure, social physique anxiety, weight control self-efficacy, and sports participation and integrates media internalized pressure, social physique anxiety, and weight control self-efficacy as personality systems to examine their effects on sports participation, to reveal the relevant mechanisms affecting college students’ sports participation and better promote the development of health. This will help to reveal the mechanisms that affect college students’ sports participation and better promote the development of healthy and confident habits among college students, thus providing practical references for enhancing their physical and mental health. Thus, the following hypotheses are proposed: (1) social physique anxiety mediates media internalized pressure and sports participation; (2) weight control self-efficacy mediates media pressure and sports participation; and (3) social physique anxiety and weight control self-efficacy act as a chain mediator between media internalized pressure and sports participation.

## Subjects and Methods

### Subjects

The survey was conducted with students enrolled in Southwestern University in China. First, the students were classified according to subject categories and grades, and then 1,500 students were randomly selected, and 375 paper questionnaires were sent out to each grade. With the help of public physical education teachers and college counselors and the approval of the Ethics Committee, the selected subjects were investigated anonymously, and all those who completed the questionnaire were unpaid volunteers. A total of 1,500 questionnaires were distributed, and 1,194 valid questionnaires were recovered. The effective recovery rate of the questionnaire was 79.6%. Among them, 306 questionnaires were excluded because of missing questions (59), not carefully filling in the questionnaire (99), and not returning the questionnaires (148).

The basic sample information is as follows (shown in Table 1): the participants include 649 male students and 545 female students with an average age of 21.31 ± 3.76 years, height of 177.71 ± 8.37 cm, weight of 68.13 ± 12.78 kg, and body mass index (BMI) of 22.74 ± 3.45. Among them, there are 297 students in freshman, 317 in sophomore, 278 in junior, and 302 in senior. The individual BMI values were converted according to BMI formula: BMI = weight (kilograms)/height (square meters). According to college students’ physical fitness BMI standard published by the Ministry of Education (Chen et al., 2019), the authors divided them into three groups by BMI: lightweight group (BMI < 20 kg/m^2^) with 309 samples, normal-weight group (BMI = 20–25 kg/m^2^) with 651 samples, and overweight group (BMI > 25 kg/m^2^) with 234 samples.

**TABLE 1:** Basic information of the sample (*N* = 1,194).

**Variable**	**Mean ± SD**	**Variables**	**Category**	***n***	**%**
Age (years)	21.313.76	Gender	Male	649	54.36
Height (cm)	177.718.37		Female	545	45.64
Weight (kg)	68.1312.78	Grade	Freshman	297	24.87
BMI (kg/m^2^)	22.743.45		Sophomore	317	26.55
			Junior	278	23.28
			Senior	302	25.29
		BMI	Lightweight group	309	25.88
			Normal-weight group	651	54.52
			Overweight group	234	19.60

*BMI, body mass index. BMI = body weight (units in kilograms)/height (units in square meters). Lightweight BMI < 20 kg/m^2^, normal BMI: 20–25 kg/m^2^, overweight BMI > 25 kg/m^2^ (BMI grouping according to the Ministry of Education of China criteria for college students).*

### Research Methods

#### Questionnaires Design and Reliability and Validity of the Questionnaire

A structured questionnaire was taken as the research tool, and the final questionnaire was completed through the preliminary draft, pretest, and analysis of pretest results based on the reference to a large number of research documents. The questionnaire is divided into five parts.

1. Personal background information: This component covers the basic background information on the subject’s sex, grade, height, weight, BMI, etc.
2. Sports participation scale: A three-question testing method according to Fox (1999) was developed, sports participation level = sports frequency × (average sports intensity + sports duration). The scores range between 2 and 72. The higher the scores, the higher is the degree of sports participation. The sports frequency is determined by the weekly sports times, using numbers 1–6 to represent “0 time/week,” “1 time/week,” “2 times/week,” “3 times/week,” “4 times/week,” and “no less than 5 times/week,” respectively. Sports duration is determined by the average time spent on each sport, using numbers 1–6 to represent “0–10 min/time,” “11–20 min/time,” “21–30 min/time,” “31–40 min/time,” “41–5 0min/time,” and “≥ 51 min/time,” respectively. Average sports intensity is determined by the fatigue level of the subjects after each sport, using numbers 1–6 to represent “very easy,” “kind of easy,” “easy,” “kind of tired,” “very tired,” and “very fatigue,” respectively. The questionnaire pretest shows high retest reliability and the correlation coefficient *r* = 0.89.
3. The scale of media internalized pressure: Mainly refer to a total of 16 items of media internalized subscales and media pressure subscales in *Sociocultural Attitudes Towards Appearance Questionnaire-3* edited by Thompson et al. (2004). The scale contains two dimensions, which are compiled by the Likert five-point scale. The options “Totally Agree,” “Very Agree,” “Not Sure,” “Very Disagree,” and “Totally Disagree” count as 5, 4, 3, 2, and 1 scores, respectively, in which questions 2, 10, and 11 are reversed questions and will be scored in reverse. The higher the total scores, the greater is the media internalized pressure perceived by individuals. Make factor analysis on the media internalized pressure scale and the predicting results show that two common factors can be extracted after the direct oblimin of 16 items, and the progressive contribution rate of the two common factors is up to 47.80%. The verification results of the measurement model of the scale are as follows: the parameter values of X^2^/DF = 1.973; adjusted goodness-of-fit index (AGFI), comparative fit index (CFI), Tucker–Lewis index (TLI), incremental fit index (IFI), goodness-of-fit index (GFI), and root mean square error of approximation (RMSEA) are, respectively, 0.985, 0.995, 0.991, 0.995, 0.994, and 0.029, which have met the acceptable standards and show that the model fits well with the data obtained from the survey and the scale has a good structure validity. The two dimensions are composed of media internalization (nine items, the higher the scores, the greater is the influence of media internalization) and media pressure (seven items, the higher the scores, the greater is the media pressure.). Cronbach’s α values of the whole media internalized pressure scale and all of its dimensions are greater than 0.70 (0.75–0.86), which fully confirms the good reliability of the scale (see Table 2). Therefore, it can be seen that the media internalized pressure scale has good reliability and validity.
4. Social physique anxiety scale: Mainly refer to the Social Physique Anxiety Scale edited by Xu (2007) with a total of 15 questions. The scale contains three dimensions, which are compiled by the five-point Likert scale. The options “Totally Agree,” “Very Agree,” “Not Sure,” “Very Disagree,” and “Totally Disagree” count as 5, 4, 3, 2, and 1 scores, respectively, in which questions 1, 3, 7, 11, 13, and 14 are reversed questions and will be scored in reverse. The higher the total scores, the higher is the social physique anxiety. Make factor analysis on the social physique anxiety scale and the predicting results show that three common factors can be extracted after the direct oblimin of 15 items, and the progressive contribution rate of the three common factors is up to 53.22%. The verification results of the measurement model of the scale are as follows: the parameter values of X^2^/DF = 1.688; AGFI, CFI, TLI, IFI, GFI, and RMSEA are, respectively, 0.989, 0.997, 0.994, 0.997, 0.996, and 0.022, which have met the acceptable standards and show that the model fits well with the data obtained from the survey, and the scale has a good structure validity. The three dimensions are composed of the worry about others’ negative evaluation (six items, the higher the scores, the higher is the worry about others’ negative evaluation), the discomfort with self-physical performance (six items, the higher the scores, the higher is the discomfort with self-physical performance), and the anxiety about social comparison (three items, the higher the scores, the higher is the anxiety about social comparison). Cronbach’s α values of the whole social physique anxiety scale and all of its dimensions are greater than 0.70 (0.71–0.88), which fully confirm the good reliability of the scale (see Table 2). Therefore, it can be seen that the social physique anxiety scale has good reliability and validity.
5. Weight control self-efficacy scale: Mainly refer to the Weight Control Self-Efficacy (WCS) compiled by Sallis, Liao, Lin, and Su et al. (Sallis et al., 1988; Yung, 2016; Chih and Chia, 2018; Chin and Chih, 2019) and revised according to the characteristics of college students to get 21 questions finally. The scale contains two dimensions, which are compiled by the five-point Likert scale. The options “Very Sure of It,” “Sure of It,” “Probably Sure of It,” “Not Sure of It,” and “Very Unsure of It” count as 5, 4, 3, 2, and 1 scores, respectively. The higher the total scores, the better is the weight control self-efficacy of an individual. Make factor analysis on the weight control self-efficacy scale and the predicting results show that the two common factors can be extracted after the direct oblimin of 21 items, and the progressive contribution rate of the three common factors is up to 52.61%. The verification results of the measurement model of the scale are as follows: the parameter values of X^2^/DF = 1.472; AGFI, CFI, TLI, IFI, GFI, and RMSEA are, respectively, 0.991, 0.998, 0.994, 0.998, 0.997, and 0.018, which have met the acceptable standards and show that the model fits well with the data obtained from the survey and the scale has a good structure validity. The two dimensions are composed of dietary self-efficacy (10 items, the higher the scores, the higher is the dietary self-efficacy) and self-efficiency for exercise (11 items, the higher the scores, the higher is the self-efficiency for exercise.) Cronbach’s α values of the whole weight control self-efficacy scale and all of its dimensions are greater than 0.70 (0.88–0.93), which fully confirm the good reliability of the scale (see Table 2). Therefore, it can be seen that the weight control self-efficacy scale has good reliability and validity.

**TABLE 2:** Factor extraction and reliability analysis of the three scales.

**Scale**	**KMO and Bartlett l test**	**Factor naming**	**Entry numbers**	**Eigenvalue**	**Explained variance %**	**Progressive explained variance %**	**Cronbach’s α coefficient**
Media internalized	KMO = 0.81	Media internalization	9	3.383	30.751	30.751	0.83
pressure^1^	*P* < 0.000	Media pressure	4	1.875	17.045	47.796	0.75
Social physique	KMO = 0.87	The worry about others’ negative evaluation	6	4.636	30.910	30.910	0.85
anxiety^2^	*P* < 0.000	The discomfort with self-physical performance	6	2.098	13.987	44.896	0.77
		The anxiety about social comparison	3	1.249	8.324	53.220	0.71
Weight control	KMO = 0.94	Dietary self-efficacy	10	6.935	43.342	43.342	0.88
self-efficacy^3^	*P* < 0.000	Self-efficiency for exercise	11	1.483	9.270	52.612	0.90

*^1^The verification results of the self-esteem scale: X^2^/DF = 1.973, AGFI = 0.985, CFI = 0.995, TLI = 0.991, IFI = 0.995, GFI = 0.994, RMSEA = 0.029; Cronbach’s α coefficient 0.86. ^2^The verification results of the body image scale were X^2^/DF = 1.688, AGFI = 0.989, CFI = 0.997, TLI = 0.994, IFI = 0.997, GFI = 0.996, RMSEA = 0.022; Cronbach’s α coefficient 0.88. ^3^The verification results of the self-efficacy scale were X^2^/DF = 1.472, AGFI = 0.991, CFI = 0.998, TLI = 0.994, IFI = 0.998, GFI = 0.997, RMSEA = 0.018; Cronbach’s α coefficient 0.93.*

#### Statistical Analysis

Data obtained in this study were analyzed using SPSS19.0 and AM0S21.0 software packages. The normal distribution of all variables was tested using the Kolmogorov–Smirnov test, and all continuous variables follow the normal distribution. The statistical methods included descriptive statistics, independent sample *t*-test, one-way analysis of variance, exploratory factor analysis, correlation analysis, structural equation model, Bootstrap analysis, etc. The significance level of all variables was set as α = 0.05.

Statistics obtained from questionnaires may lead to common method biases. This research uses Harman single factor test to test the possible common method biases (Zhou and Long, 2004). The results show that the characteristic roots of a total of 19 factors are greater than 1, among which the largest factor explained variance is 15.81%, far from the critical standard of 40%. It can be seen that the research is less likely to be affected by common method biases, which is within the acceptable range.

## Results

### Comparison of Differences in Media Internalized Pressure, Social Physique Anxiety, Weight Control Efficacy, and Sports Participation by Personal Background in College Students

Table 3 shows the following:

**TABLE 3:** Comparison of differences in media internalized pressure, social physique anxiety, weight control efficacy, and sports participation by personal background in college students (*N* = 1,194).

**Variables**	**MI**	**MP**	**NE**	**SP**	**SC**	**DSE**	**SEE**	**SPN**
**Gender**								
Male	25.01(6.19)	19.58(4.02)	16.30(3.94)	17.89(2.85)	7.69(2.29)	26.58(16.14)	31.63(9.58)	18.33(9.78)
Female	26.74(5.39)	23.05(4.08)	17.33(3.72)	18.32(2.79)	8.30(2.14)	23.07(7.49)	25.45(9.50)	12.84(7.29)
*t*-value	−3.82***	−10.97***	−3.51***	−1.98*	−3.60***	3.74***	8.37***	8.41***
**Grade**								
Freshman^1^	25.84 (5.31)	22.59 (4.12)	17.35 (3.56)	18.45 (2.60)	8.32 (2.14)	23.67 (17.68)	27.30 (9.63)	13.74 (8.24)
Sophomore^2^	25.84 (6.08)	22.17 (4.51)	16.66 (3.99)	17.99 (2.91)	7.87 (2.28)	24.93 (8.18)	28.64 (9.83)	16.18 (9.12)
Junior^3^	27.82 (5.99)	23.61 (4.35)	16.20 (3.99)	17.80 (3.10)	7.77 (2.17)	24.68 (7.39)	28.59 (12.59)	15.32 (9.10)
Senior^4^	26.04 (5.71)	21.97 (4.42)	16.88 (3.94)	17.67 (2.97)	8.17 (2.14)	24.21 (6.95)	29.88 (10.80)	16.92 (9.79)
*F*-value	1.58	2.39	2.03	1.71	2.22	0.71	1.12	4.00**
LSD								1<4; 2<4
**BMI**								
Lightweight^1^	25.77 (5.79)	21.54 (4.16)	16.69 (3.76)	18.08 (2.77)	8.08 (2.11)	24.26 (14.99)	29.46 (9.78)	16.33 (9.30)
Normal-weight^2^	26.37 (5.73)	21.76 (4.52)	17.25 (3.83)	18.21 (2.72)	8.12 (2.30)	24.58 (8.28)	31.95 (11.83)	17.65 (10.53)
Overweight^3^	24.76 (6.09)	18.79 (3.73)	15.88 (4.16)	18.35 (3.77)	7.19 (2.23)	26.51 (7.69)	26.68 (9.68)	14.20 (8.14)
*F*-value	1.80	9.05***	3.28*	0.30	3.46*	0.66	9.51***	6.34**
LSD		1<2; 2>3	1<2; 2>3		1<2; 2>3		1<2; 1>3; 2>3	1<2; 1>3; 2>3

*P < 0.05; **P < 0.01; ***P < 0.001. *t*-value, the value of the independent-sample *t*-test; *F*-value, the value of the one-way ANOVA test; LSD, least square difference. MI, media internalization; MP, media pressure; NE, the worry about others’ negative evaluation; SP, the discomfort with self-physical performance; SC, the anxiety about social comparison; DSE, dietary self-efficacy; SEE, self-efficiency for exercise; SPN, sports participation.

1. There were significant sex differences among media internalization (*T* = −3.82, *P* < 0.001), media pressure (*T* = −10.97, *P* < 0.001), the worry about others’ negative evaluation (*T* = −3.51, *P* < 0.001), the discomfort with self-physical performance (*T* = −1.98, *P* < 0.05), the anxiety about social comparison (*T* = −3.60, *P* < 0.001), dietary self-efficacy (*T* = 3.74, *P* < 0.001), self-efficiency for exercise (*T* = 8.37, *P* < 0.001), and sports participation (*T* = 8.41, *P* < 0.001). The results showed that boys scored higher than girls in three aspects of dietary self-efficacy, self-efficiency for exercise, and sports participation, but girls scored higher than boys in five aspects of media internalization, media pressure, the worry about others’ negative evaluation, the discomfort with self-physical performance, and the anxiety about the social comparison.
2. (2) There were significant grade differences in sports participation (*F* = 4.00, *P* < 0.01), whereas scores in these seven aspects of media pressure (*F* = 2.39, *P* > 0.05), media internalization (*F* = 1.58, *P* > 0.05), the worry about others’ negative evaluation (*F* = 2.03, *P* > 0.05), the discomfort with self-physical performance (*F* = 1.71, *P* > 0.05), the anxiety about social comparison (*F* = 2.22, p > 0.05), dietary self-efficacy (*F* = 0.71, *P* > 0.05), and self-efficiency for exercise (*F* = 1.12, *P* > 0.05) were not related to grade. The results showed that the senior students score significantly higher than the freshmen and sophomores on sports participation.
3. Media pressure (*F* = 9.05, *P* < 0.001), the worry about others’ negative evaluation (*F* = 3.28, *P* < 0.05), the anxiety about social comparison (*F* = 3.46, *P* < 0.05), self-efficiency for exercise (*F* = 9.51, *P* < 0.001), and sports participation (*F* = 6.34, *P* < 0.01) were significantly affected by BMI, but media internalization (*F* = 1.80, *P* > 0.05), the discomfort with self-physical performance (*F* = 0.30, *P* > 0.05), and dietary self-efficacy (*F* = 0.66, *P* > 0.05) did not seem to be associated with BMI. The results showed that the normal-weight group was significantly higher than the lightweight group, and the overweight group in media pressure, the worry about others’ negative evaluation, and the anxiety about social comparison and the overweight group were significantly lower than the normal weight group and the overweight group in self-efficiency for exercise and sports participation.

### Related Analysis on College Students’ Media Internalized Pressure, Social Physique Anxiety, Weight Control Self-Efficacy, and Sports Participation

Pearson correlation was used to analyze the correlation coefficients among media internalized pressure, social physique anxiety, weight control self-efficacy, and sports participation (see Table 4). The results showed that media internalized pressure, social physique anxiety, weight control self-efficacy, and sports participation were positively correlated. Therefore, the correlation between variables is significant, which can provide a good basis for the subsequent exploration of mediating effect test.

**TABLE 4:** Related analysis on college students’ media internalized pressure, social physique anxiety, weight control self-efficacy, and sports participation (*N* = 1,194).

	***M* ± SD**	**MI**	**MP**	**NE**	**SP**	**SC**	**DSE**	**SEE**	**SPN**
MI	21.494.38	1.00							
MP	25.965.79	0.56**	1.00						
NE	16.883.83	0.22**	0.24**	1.00					
SP	18.142.82	0.21**	0.17**	0.51**	1.00				
SC	8.042.21	0.27**	0.19**	0.48**	0.45**	1.00			
DSE	24.5812.18	0.31**	0.35**	0.28**	0.24**	0.21**	1.00		
SEE	28.2410.01	0.36**	0.38**	0.34**	0.29**	0.24**	0.49**	1.00	
SPN	15.348.91	0.48**	0.45**	0.47**	0.43**	0.41**	0.35**	0.51**	1.00

*P < 0.05; **P < 0.01.

### College Students’ Model Validation Analysis

**FIGURE 1:**
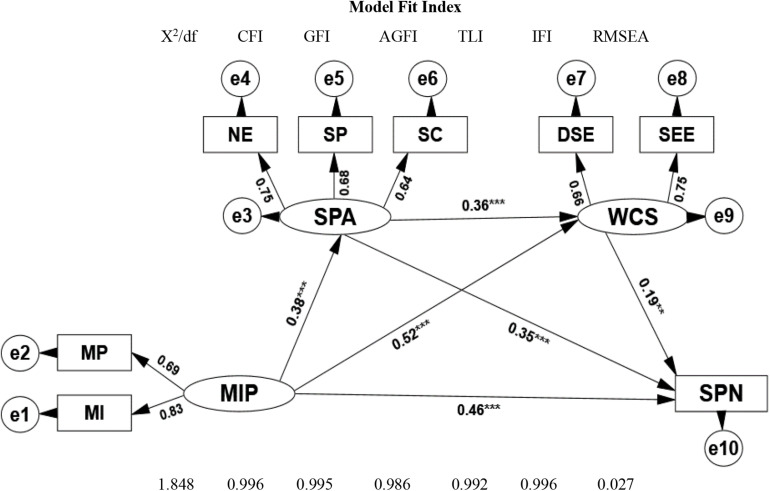
Path analysis diagram and model fit test of media internalized pressure, social physique anxiety, weight control self-efficacy, and sports participation. MIP, media internalized pressure; SPA, social physique anxiety; WCS, weight control self-efficacy; SP, sports participation; MP, media pressure; MI, media internalization; NE, the worry about others’ negative evaluation; SP, the discomfort with self-physical performance; SC, the anxiety about social comparison; DSE, dietary self-efficacy; SEE, self-efficiency for exercise; SPN, sports participation. *P < 0.05; **P < 0.01; ***P < 0.001.

To investigate the relationship between media internalized pressure and social physique anxiety, weight control self-efficacy, and sports participation and examine the intermediary role of social physique anxiety and weight control self-efficacy and according to the intermediary effect testing process proposed by Wen and Ye (2014), this research adopts AMOS to make a structural equation model analysis on the relationship among media internalized pressure, social physique anxiety, weight control self-efficacy, and sports participation. Take for example Figure 1 after the model has been modified, and the model fit indexes are: X^2^/DF = 1.848 < 2.000, CFI = 0.995, GFI = 0.995, AGFI = 0.986, TLI = 0.992, and IFI = 0.996, all of which are >0.900 and RMSEA = 0.027 < 0.080, which shows that the model can be built up.

It is easy to find from the standardized path coefficient β and significance level in the mixed model structure in Figure 1 that media internalized pressure has a significantly positive predictive effect on social physique anxiety, weight control self-efficacy, and sports participation (β = 0.38^∗∗∗^, *P* < 0.001; β = 0.52^∗∗∗^, *P* < 0.001; β = 0.46^∗∗∗^, *P* < 0.001); meanwhile, social physique anxiety has a significantly positive predictive effect on weight control self-efficacy and sports participation (0.36^∗∗∗^, *P* < 0.001; β = 0.35^∗∗∗^, *P* < 0.001), and weight control self-efficacy has a significantly positive predictive effect on sports participation (β = 0.19^∗∗^, *P* < 0.01). To verify the intermediary effect of social physique anxiety and weight control self-efficacy on media internalized pressure and sports participation, the study adopts a non-parametric percentile Bootstrap process to make a significance test on intermediary effect. Five thousand samples were taken repeatedly from the original data to calculate a 95% confidence interval. If the standardized path coefficient 95% CI does not include 0, that means the intermediary effect is significant. The 95% CI of the chained intermediary effect of media internalized pressure from media internalized pressure to social physique anxiety, weight control self-efficacy, and sports participation is 0.011–0.035; that of media internalized pressure from social physique anxiety to sports participation is 0.118–0.166; that of media internalized pressure from weight control self-efficacy to sports participation is 0.092–0.132. The three intervals mentioned earlier do not include 0, which shows each intermediary effect is significant (see Table 5).

**TABLE 5:** Path and effect decomposition table of media internalized pressure on sports participation.

**Effect**	**Path relationship**	**Effect size**	**Bootstrap SE**	**Bootstrap 95% CI**
Direct effect	Media internalized pressure → sports participation	0.456	0.041	0.382–0.546
Indirect effect	Media internalized pressure → social physique anxiety → weight control self-efficacy → sports participation	0.027	0.006	0.011–0.035
	Media internalized pressure → social physique anxiety → sports participation	0.136	0.018	0.118–0.166
	Media internalized pressure → weight control self-efficacy → sports participation	0.102	0.012	0.092–0.132
Total intermediary effect		0.265	0.024	0.221–0.317

*SE, standard error; CI, confidence interval.*

## Discussion

### From the Perspective of the Relationship of Personal Background With Media Internalized Pressure, Social Physique Anxiety, Weight Control Efficacy, and Sports Participation in College Students

This study found that girls in college are more likely to feel the influence of internalization, media pressure, the worry about others’ negative evaluation, the discomfort with self-physical performance, and the anxiety about social comparison than boys, whereas boys pay more attention to dietary self-efficacy, self-efficiency for exercise, and sports participation than girls. The results show that girls pay more attention to their body shape and appearance than boys, which is easy to produce media internalized pressure and social physique anxiety, whereas boys are more self-discipline than girls, and they are better than girls in weight control efficiency and sports participation. This result is consistent with previous views. Social culture regards girl’s “thin for beauty” as the value advocated by the media, which is easy to make girls internalize the ideal image of “Slim and Energetic,” thus causing media pressure and social physique anxiety (Wang et al., 2016; Liu and Wang, 2019; Xu and Gao, 2019), whereas boys pay more attention to masculinity, and they will consider increasing muscle content (Renée, 2003; Zhao et al., 2017). At the same time, boys are better than girls in physical fitness and motor skills, and boys’ relative self-discipline weight control efficiency is better than girls, and their sports participation is also better than girls (Hou and Yang, 2017; Hu, 2017).

This study also found that seniors were more likely to participate in sports than juniors because they have less academic workload and more disposable time, and the pressure of employment forces them to participate in sports to adjust their body and mind (Ouyang et al., 2020). Therefore, senior students have more advantages in sports participation.

In addition, this study also found significant differences between BMI and media pressure, the worry about others’ negative evaluation, the anxiety about social comparison, weight control efficacy, and sports participation. The normal-weight group is more likely to be affected by media pressure, the worry about others’ negative evaluation, the anxiety about social comparison, weight control efficacy, and sports participation than the other two groups. Does this indicate that the normal-weight group is more likely to be affected by media pressure? Also, the worry about others’ negative evaluation leads to anxiety about social comparison and then improves weight control efficacy. This result is consistent with previous studies (Yea and Yea, 2014; Li and Zhou, 2016; Meng et al., 2017).

### From the Perspective of Structured Path Model of Media Internalized Pressure, Social Physique Anxiety, Weight Control Efficacy, and Sports Participation

#### Influence of Media Internalized Pressure on Sports Participation

This research finds that media internalized pressure has a significant positive correlation with sports participation. This result indicates that the greater the media internalized pressure, the higher is the degree of sports participation and *vice versa*. This is basically consistent with the research of Wang and Zhang (2012) and Hang and Deng (2017). College students are easy to be exposed under pressure from social culture, and the media promotes “slim body” and “perfect body,” which can greatly enhance their internalization of “slim body” and “perfect body” and have negative effects on body satisfaction; thus, they take the standard publicized by the media as a guide to change their own criteria to meet the standard (Menzel et al., 2011; Wei et al., 2012; Jackson and Chen, 2015; Huang et al., 2020). Simultaneously, research also shows that the higher the internalized degree of social culture, the more students pay attention to their appearance. As the “ideal body” advocated by the media has become a part of college students’ self-identification, the higher the self-identification with “ideal body,” the greater is the gap between their ideal ego and real ego, which will lead to an increase in body dissatisfaction and the act of changing their body shape to improve their body image (Stice et al., 1996; Bessenoff, 2016). At the same time, some scholars also believe that media internalization of ideal body shape and media pressure will lead to a decrease in their body satisfaction (Chen et al., 2010; Li et al., 2015; Wang et al., 2016; Ma et al., 2020), to bring all kinds of motivation to improve physical appearance (for example, diet control and exercise) to remove the negative body image (Wei et al., 2012; Gillen, 2015; Andrew et al., 2016). In addition, body image has a positive correlation with sports participation. College students’ improving their body image can also promote their sports participation and *vice versa* (Ouyang et al., 2020). Therefore, media internalized pressure is an important factor affecting college students’ sports participation, and there is a positive predictive effect between the two.

#### Intermediary Effect of Social Physique Anxiety

This research finds that media internalized pressure has a significant positive correlation with social physique anxiety. This result indicates that people with higher media internalized pressure tend to have higher social physique anxiety, whereas those with lower media internalized pressure tend to have lower social physique anxiety. This result is basically consistent with the findings of the research of Harrison (2010) and Sharp et al. (2014): social media creates a new esthetic standard, which will increase media internalized pressure. The longer people read or watch, the greater is the psychological pressure and negative body image, which will then generate social physique anxiety. Meanwhile, it proves the research findings of Dittmar (2009); Halliwell and Dittmar (2005), and Yang et al. (2020): the large media internalized pressure will lead to the comparison between ideal body shape and real body shape, which will cause the body image dissatisfaction and increase social physique anxiety. This research also finds that social physique anxiety has a significant positive correlation with sports participation. This result shows that college students’ sports participation will increase as the social physique anxiety of individuals increases. These research results have just proved those of Eklund and Crawford (1994); Hausenblas and Martin (2000), and He (2018): social physique anxiety can boost the degree of sports participation, and it is also one of the motivations of sports participation. In addition, this research also finds that media internalized pressure can not only directly and positively affect sports participation but also indirectly and positively affect sports participation through the intermediary role of social physique anxiety. Some scholars believe that media internalized pressure can not only positively influence college students’ sports participation but also positively influence their sports participation through social physique anxiety (Tang and Ji, 2011; Wang et al., 2016; Hu, 2017). In conclusion, combining with the Bootstrap test program for intermediary effect, it can be inferred that social physique anxiety plays an intermediary role between media internalized pressure and sports participation is true.

#### Intermediary Effect of Weight Control Self-Efficacy

This research finds that media internalized pressure has a significant positive correlation with weight control self-efficacy. This result indicates that the greater the media internalized pressure, the better is the weight control self-efficacy and *vice versa*. This is consistent with the findings of Chen and Lin (Chen et al., 2009; Chih and Chia, 2018) that media internalized pressure has a positive predictive effect on weight control self-efficacy. This research also finds that weight control self-efficacy has a significant positive correlation with sports participation. This result shows that an individual’s sports participation will increase as their weight control self-efficacy is getting better. Some research points out that self-efficacy has a positive correlation with college students’ sports participation (Sun, 2010; Koring et al., 2012; Parschau et al., 2013; Du et al., 2016). The higher the self-efficacy, the higher is the degree of sports participation. On the contrary, the lower the self-efficacy, the lower is the degree of sports participation. Meanwhile, some researchers also point out that weight control self-efficacy is a direct factor affecting sports participation and has a good positive predictive effect on sports participation (Kuan and Huang, 2012; Yea and Yea, 2014; Meng et al., 2017; Annesi, 2019; Shang and Ku, 2019). This research also finds that media internalized pressure can not only directly and positively affect sports participation but also indirectly and positively affect sports participation through the intermediary role of weight control self-efficacy. Media internalized pressure can have a negative effect on people’s image satisfaction. Once it is reduced, people will enhance their weight control behavior (Hsin and Hung, 2008; Sun et al., 2013; Cao et al., 2014); meanwhile, this improves the weight control self-efficacy (Chen et al., 2009; Chih and Chia, 2018), which can effectively improve college students’ sports participation. In conclusion, combining with the Bootstrap test program for intermediary effect, it can be inferred that weight control self-efficacy plays an intermediary role between media internalized pressure and sports participation is true.

### Chained Intermediary Effect of Social Physique Anxiety and Weight Control Self-Efficacy

This research shows through the results of the Bootstrap test program for the intermediary effect that media internalized pressure can affect college students’ sports participation through social physique anxiety, which affects through part of the intermediary effect of weight control self-efficacy. This verifies Hypothesis 3 that social physique anxiety and weight control self-efficacy play a chained intermediary role between media internalized pressure and sports participation, which shows that people with higher media internalized pressure are more likely to have higher social physique anxiety, leading to higher weight control self-efficacy and even weight control behaviors, to promote the college students’ sports participation. On the contrary, college students with lower media internalized pressure tend to have lower social physique anxiety and weight control self-efficacy, as well as lower sports participation. The research discussed earlier has proven that social physique anxiety and weight control self-efficacy act, respectively, as the intermediary of media internalized pressure and sports participation, and all of the previous researches have confirmed the correlation between social physique anxiety and weight control self-efficacy (Shu et al., 2011; Annesi and Mareno, 2015; Yuan et al., 2016). That is to say, people with higher social physique anxiety have a positive correlation on the individual’s weight control self-efficacy, whereas those with lower social physique anxiety have a negative effect on the individual’s weight control self-efficacy. Therefore, media internalized pressure can positively affect college students’ sports participation through social physique anxiety and weight control self-efficacy.

In a word, media internalized pressure can not only directly affect sports participation but also affect sports participation through indirect paths such as social physique anxiety and the chained intermediary effect of social physique anxiety–weight control self-efficacy. This provides a theoretical basis for college students to improve their sports participation and also provides an effective practical reference to know college students’ media internalized pressure, social physique anxiety, and weight control self-efficacy to improve their sports participation.

## Conclusion and Limitation

### Conclusion

Media internalized pressure has a significantly positive predictive effect on social physique anxiety, weight control self-efficacy, and sports participation; social physique anxiety has a significantly positive predictive effect on weight control self-efficacy and sports participation, and weight control self-efficacy also has a significantly positive predictive effect on sports participation; meanwhile, social physique anxiety and weight control self-efficacy can have an intermediary effect on media internalized pressure and sports participation, and “social physique anxiety + weight control self-efficacy” also have a chained intermediary effect on media internalized pressure and sports participation.

It is hoped that teachers in higher education institutions can understand more about the psychological characteristics of college students by reading this paper and develop more reasonable exercise participation programs based on these psychological characteristics, for example, reasonable sports participation programs based on BMI classification, preferred sports participation programs based on sex characteristics and adapted sports participation programs based on psychological status. By promoting the sports participation of college students, the physical and mental health of college students will be better improved.

### Limitation

The shortcomings of this research are the following: this is a cross-sectional research, and we hope that research in the future can consider verifying the results of this research by using the method of follow-up research. In addition, the participants of this research are the students of a Southwest University in China. We hope that we can select a wider range of participants for future research to test the external validity of the research results.

## Data Availability Statement

The original contributions presented in the study are included in the article/Supplementary material, further inquiries can be directed to the corresponding author/s.

## Ethics Statement

The studies involving human participants were reviewed and approved by the Ethics Committee of Southwest University Hospital. The patients/participants provided their written informed consent to participate in this study.

## Author Contributions

YO and JioL carried out the protocol and questionnaire survey. JT recruited the students enrolled in Southwestern University. KW and JinL undertook the statistical analysis and graphical representation of the data. YO and JioL revised the draft. All authors designed this study, contributed to the article, and approved the final manuscript.

## Conflict of Interest

The authors declare that the research was conducted in the absence of any commercial or financial relationships that could be construed as a potential conflict of interest.
